# Making Bunyaviruses Talk: Interrogation Tactics to Identify Host Factors Required for Infection

**DOI:** 10.3390/v8050130

**Published:** 2016-05-13

**Authors:** Amber M. Riblett, Robert W. Doms

**Affiliations:** 1 Department of Microbiology, Perelman School of Medicine, University of Pennsylvania, Philadelphia, PA 19104, USA; ariblett@mail.med.upenn.edu; 2 Department of Pathology and Laboratory Medicine, Children’s Hospital of Philadelphia, Philadelphia, PA 19104, USA

**Keywords:** bunyavirus, high-throughput screening, host-pathogen interaction, haploid genetic screening, RNAi screening, yeast two-hybrid, affinity purification mass spectrometry

## Abstract

The identification of host cellular genes that act as either proviral or antiviral factors has been aided by the development of an increasingly large number of high-throughput screening approaches. Here, we review recent advances in which these new technologies have been used to interrogate host genes for the ability to impact bunyavirus infection, both in terms of technical advances as well as a summary of biological insights gained from these studies.

## 1. Introduction

Viruses rely on a large number of host cellular factors and pathways as they enter, traffic through, replicate, assemble, and exit the cell. Identification of host cell factors needed for virus replication, such as cell surface receptors, has provided tremendous insights into viral lifecycles and pathogenesis. Traditionally, relatively reductionist approaches have been taken to identify specific interactions between viral and host cell molecules. More recently, rapid advances in high-throughput screening technologies based upon small molecules, loss-of-function libraries, and interactome characterization have informed our understanding of nearly every stage of virus replication cycles and identified valuable targets for antiviral therapeutics. Since the technical aspects for most of these techniques have been extensively reviewed, we aim here to present a summary of their use within the bunyavirus field, a brief comparison of their relative advantages and disadvantages, technical considerations that apply to screening with bunyaviruses, and recent advances in screening approaches that may be of general interest. The emergence and spread of newly-identified bunyaviruses in recent years, as well as important progress toward a more detailed understanding of bunyavirus structure and genetics, has renewed interest in this large and diverse family of viruses. Simultaneous advances in genome-wide screening techniques, and their well-demonstrated power to identify novel host cellular factors that either support or restrict viral infection, present an exciting tool for probing many aspects of bunyavirus biology.

## 2. Genetic Approaches to Identify Bunyavirus Host Factors

RNA interference (RNAi) technology was the first of a new generation of high-throughput screening approaches applied to the study of virus-host interactions. Examples of its use include the pioneering screens by Cherry *et al.* to uncover a role for host organelle-reshaping and ribosomal proteins in *Drosophila* C virus (DCV) replication [1,2], a series of 2008 studies from multiple labs that identified many host factors necessary for human immunodeficiency virus (HIV)-1 replication [3,4,5], and the characterization in 2009 by Brass *et al*. of interferon-induced transmembrane (IFITM) proteins as restriction factors for influenza, West Nile, and dengue viruses [6]. For this screening technique, the incorporation of small interfering RNAs (siRNAs) into the RNA-induced silencing complex (RISC) effects the cleavage of target cellular mRNA and consequent knockdown of gene product expression. These siRNAs can be either directly introduced into the cell, or derived from supplied precursors: long double-stranded RNAs (dsRNAs) or short hairpin RNAs (shRNAs) that are then processed by cellular machinery. The availability of increasingly robust genome-wide libraries for RNAi screening has greatly increased its popularity as a high-throughput, unbiased screening platform.

Within the bunyavirus field, a 2013 RNAi screen by Hopkins *et al.* in *Drosophila* cells used dsRNAs targeting more than 13,000 genes, identifying 124 that restricted infection by the phlebovirus Rift Valley fever virus (RVFV), with genes involved in DNA replication, the cell cycle, and mRNA metabolic processing being significantly enriched [7]. Among these were the catalytic component of the mRNA decapping machinery mRNA-decapping enzyme 2 (Dcp2), as well as two decapping activators, DEAD (Asp-Glu-Ala-Asp) Box Helicase 6 (DDX6) and U6 snRNA-associated Sm-like protein LSm7 (LSM7). Bunyaviruses “cap-snatch” the 5′ ends of host mRNAs, and the authors showed that RVFV specifically cap-snatches the 5′ ends of Dcp2-targeted mRNAs, as did La Crosse virus (LACV), a member of the *Orthobunyavirus* genus. The year after, Meier *et al.* performed a screen using Uukuniemi virus (UUKV) in HeLa cells expressing the surface lectin CD209, which is an attachment factor for UUKV in dendritic cells [8]. Two independent genome-wide siRNA libraries were used from two manufacturers: one with four unpooled siRNAs per gene and one with four pooled siRNAs per gene. In both screens the vesicle-soluble NSF attachment protein receptor (vSNARE) vesicle-associated membrane protein 3 (VAMP3) was identified as a host factor required for the entry of UUKV. The importance of VAMP3 was also indicated by virtue of its being a target for the endogenous microRNA miR-142-3p, a microRNA identified as impacting infection after analysis of the seed sequences of the siRNAs used for screening. The authors examined incoming UUKV virions trafficking through the endocytic pathway and noted increasing colocalization of virions with VAMP3 as they moved within vesicles through the cytoplasm. At 20 min after internalization, maximum colocalization between UUKV virions and VAMP3 was observed within vesicles positive for lysosomal-associated membrane protein 1 (LAMP1), a marker for late endosomes and lysosomes. In VAMP3-depleted cells, incoming virions failed to reach these LAMP1-positive vesicles, indicating that their trafficking was arrested at an earlier endosomal compartment. In contrast, depletion of VAMP3 did not affect the entry of Semliki Forest virus (SFV), which penetrates from early endosomes [9], or of influenza A virus (IAV), which fuses from late endosomes [9,10,11]. This suggests that the entry defect of UUKV in the absence of VAMP3 is not due to a lack of endosomal acidification, and that late steps of viral entry of UUKV are distinct from those of IAV. Interestingly, VAMP3 has been shown to be required for the fusion of multivesicular bodies with autophagosomes [12], although it is unclear whether this activity may have any bearing on its role in bunyavirus entry. These data informed our understanding of the host cellular machinery required for maturation of endosomal compartments and for the fusion of late-penetrating viruses within the acidic environment of late endosomes.

The arrival of haploid screening in human cells, described by Carette *et al*. in 2009, offered a loss-of-function forward genetic approach as a powerful alternative to traditional siRNA-based depletion screens [13,14]. In these screens, null alleles are generated in mammalian haploid cells using insertional mutagenesis, and the resulting cellular library is challenged by a selective agent such as a virus or toxin. Surviving cells, which presumably lack a gene required by the selective agent as a consequence of retroviral insertion, are pooled and deep sequencing is used to map the insertion sites of the mutagenizing lentivirus. Statistically enriched insertion sites within the surviving (selected) population compared to the original mutant library yield a list of genes whose disruption confers a resistance phenotype. This approach identified the homotypic fusion and vacuole protein sorting (HOPS) complex and the endo/lysosomal cholesterol transporter protein Nieman-Pick 1 (NPC1) as essential host factors for Ebola virus (EBOV) entry, and uncovered the receptor-switching process of Lassa virus (LASV) as it engages first its α-dystroglycan receptor at the cell surface and then later its intracellular receptor, LAMP1 [15,16,17]. These studies have provided potential antiviral targets, as well as insight into the molecular determinants of host tropism, for these important human pathogens.

In 2014, Petersen *et al.* used a recombinant vesicular stomatitis virus (rVSV), in which the Andes virus (ANDV) glycoproteins are expressed on the VSV core, to identify cellular host factors required for ANDV entry [18]. This rVSV-ANDV was used to challenge a human haploid mutant library and multiple members of the sterol regulatory pathway were identified as impacting ANDV entry. This dependence upon cholesterol was validated using live wild-type ANDV, a member of the New World hantaviruses that are causative agents of hantavirus pulmonary syndrome (HPS). Cholesterol requirement during viral entry was verified through the use of Chinese hamster ovary (CHO) knockout cell lines, pharmacological inhibitors, siRNA depletion, and transcription activator-like effector nuclease (TALEN) disruption of members of the cholesterol pathway, as well as by direct depletion of cholesterol in the cellular membranes. Virus binding at the cell surface was unaffected, but an internalization defect was observed within cells that lack a functional sterol regulatory pathway. The following year, Kleinfelter *et al.* independently confirmed these findings and extended the cholesterol-dependence phenotype to members of both the Old World and New World hantavirus clades [19]. Cholesterol depletion was shown to significantly delay virus internalization, and to inhibit the ability of virions to fuse with cellular membranes. This finding is intriguing, as the pH requirement for ANDV implicates it as a late-penetrating virus, but the liposome fusion results from Kleinfelter *et al.* suggest that ANDV may require a greater cholesterol concentration than what is present in the membranes of late endosomes. Detailed mechanistic studies will be needed to reconcile this, and to determine whether hantaviruses somehow modulate endosomal cholesterol composition, fuse specifically at cholesterol-rich microdomains, or whether cholesterol plays some other role during virus-membrane fusion.

We recently employed the haploid genetic screening technology using RVFV and identified a role for heparan sulfate proteoglycans as attachment factors for RVFV on some but not all cell types [20]. Within the surviving RVFV-resistant population, there was a significant enrichment of inactivating insertions into genes encoding proteins involved in nearly every step of the heparan sulfate biosynthesis pathway, as well as multiple members of the conserved oligomeric Golgi (COG) complex. The COG complex is required for normal Golgi function, and it has been shown that perturbing the COG complex leads to a defect in *O*-linked glycosylation [21,22]. Infection of heparan sulfate-deficient cells was also inhibited for a panel of pathogenic primary RVFV isolates, indicating that the use of glycosaminoglycans by RVFV was not a trait acquired during repeated passaging and laboratory strain attenuation. The screen also identified the previously-uncharacterized gene *PTAR1*, and disruption of this gene led to decreased levels of heparan sulfate on the cell surface and conferred resistance to RVFV infection. This was consistent with the results published by Blomen *et al.* showing that cells lacking protein prenyltransferase alpha subunit repeat containing 1 (PTAR1) have a defect in glycosylation [23].

## 3. Recent Advances in Genetic Screening Techniques

Bunyavirus research going forward will be greatly aided by many exciting developments in loss-of-function screening technology. In addition to the near-haploid human cell line HAP1, haploid cell lines have been generated from fish, mouse, monkey, and rat embryonic stem cells [24,25,26,27,28]. A fully haploid human cell line has also been derived by genome editing using the clustered regularly interspaced short palindromic repeats (CRISPR) RNA-guided endonuclease Cas9 to excise the fragment of chromosome 15 that was integrated onto chromosome 19 and was preventing the HAP1 cell line from being fully haploid [29]. This updated, engineered haploid cell line, termed eHAP, will likely replace the HAP1 line in the generation of new mutagenesis libraries.

CRISPR/Cas9 technology has now also been applied to high-throughput functional genomic screening. This DNA-editing technique was adapted from the type II CRISPR bacterial adaptive immune system in which the endonuclease Cas9 is recruited to the DNA of invading pathogens by two RNA components: a CRISPR RNA (crRNA) that contains a DNA fragment complementary to the foreign target, and a trans-activating CRISPR RNA (tracrRNA) which acts as a scaffold. The crRNA and tracrRNA can be fused to form a single guide RNA (sgRNA), greatly simplifying the process of synthesizing and delivering custom CRISPR/Cas9 machinery in order to disrupt a gene of interest. The Cas9-induced cleavage triggers the cell’s double-strand break repair response, leading either to indel mutations, or (if supplied) the introduction of a sequence of interest. For a detailed technical review of CRISPR/Cas systems and their utility for genome engineering, see reference [30]. 

Generation of sgRNA libraries providing genome-wide targeting by CRISPR/Cas9 has opened the door to a new method of high-throughput screening to identify host factors required for infection. In one recent study, a CRISPR sgRNA library was used to identify genes required for the induction of cell death by West Nile virus (WNV) [31]. Lentiviral vector delivery of both the sgRNAs and the Cas9 endonuclease have been developed, and are being optimized for efficient delivery [32].

## 4. Small Molecule Screening

The lack of available antivirals for bunyaviruses has renewed interest in the screening of small molecule inhibitors, including the repurposing of clinically-approved pharmacologics. In 2016, Islam *et al.* used a high-throughput drug screen to identify compounds, which potently inhibited RVFV infection, based upon a replication-competent recombinant virus lacking the gene encoding the nonstructural protein S (NSs) and bearing a fluorescent reporter [33]. This study yielded six compounds (out of approximately 28,000 screened) that exhibited inhibitory activity at low concentrations with minimal cytotoxicity. Follow-up studies will be required to determine the mechanism of action of these compounds and their potential suitability as therapeutic agents against RVFV and perhaps other bunyaviruses.

Advances in inhibitor drug screening have included methods to study the interactions between compounds that may be able to synergistically restrict viral infection. In 2012, Tan *et al.* described multiplex screening for interacting compounds (MuSIC), an analysis of all of the possible pairs of 1000 commercially available compounds that were approved by the U.S. Food and Drug Administration (FDA) or clinically tested [34]. The authors identified anti-inflammatory drugs as a group that synergistically enhanced anti-HIV activity and informed drug-interaction network formation. Such screening methods may uncover previously uncharacterized therapeutic options within the pool of clinically-tested or -approved drugs, which is particularly attractive for bunyavirus diseases, most of which lack vaccines and therapeutic options.

## 5. Biochemical Approaches: Viral Proteins as Bait for Host Factors

Valuable insight into the host-pathogen relationship can also be gleaned from interrogating physical interactions between viral and cellular proteins. The most widely-used applications for probing protein-protein interactions are yeast two-hybrid (Y2H) and affinity purification followed by mass spectrometry (AP/MS) techniques. Y2H screens utilize a reporter gene whose expression depends upon the activity of a transcription factor whose modular binding and activation domains have been fused, respectively, to bait and prey proteins. The protein of interest whose interacting partners are to be probed is the bait, and the prey proteins are typically libraries of proteins (or protein fragments) covering the genome of the organism of interest. These hybrid proteins are then introduced into cells, and if the bait and prey proteins interact, the binding and activation domains come into close enough proximity to reconstitute transcription factor activity and effect the expression of the reporter gene. For AP/MS, a bait protein of interest is pulled down via affinity for an antibody against either the protein itself or a tag to which it has been fused. Tandem affinity purification (TAP) systems are attractive approaches for their ability to reduce non-specific interactions. The classic TAP tag comprises a Protein A tag and calmodulin binding peptide (CBP) tag separated by a recognition sequence that is specific to the Tobacco etch virus (TEV) protease. Protein complexes are purified by first capturing with the terminal IgG-binding Protein A tag, then using the TEV protease to cleave and release bound complexes and expose the CBP, followed by a second affinity purification step of immobilization on calmodulin. This dual-affinity approach reduces co-purifying non-specific interactions.

A variety of protein-protein interaction approaches have been employed with the bunyavirus NSs, which is known to be critical for viral defense against the host’s type I interferon response. Léonard and colleagues performed Y2H screening of a HeLa cDNA library using the Bunyamwera virus (BUNV) NSs protein as bait [35]. They identified Mediator of RNA polymerase II transcription subunit 8 (MED8), a component of the Mediator complex, as a target of NSs during infection, and mapped this interaction to the C-terminal domain of NSs. Mediator is a key regulator of RNA polymerase II transcriptional activity, and C-terminal NSs truncation mutants were unable to effect host cell protein shutoff. Additionally, whereas wild-type BUNV is able to inhibit transcription of interferon-β (IFN-β) mRNA, infection with a recombinant BUNV lacking the MED8 interaction domain of NSs resulted in strong induction of the IFN-β promoter and thus rendered the virus sensitive to the host interferon response. The domain of NSs responsible for this MED8 interaction contains a motif that is highly conserved among orthobunyaviruses, suggesting that this interaction represents an important defense mechanism used by the virus to dismantle the host interferon response. In 2012, Rönnberg *et al.* used Y2H screening with a mouse embryo cDNA library with the hantaviruses Puumala virus (PUUV) and Tula virus (TULV) NSs proteins as bait [36]. From these two screens, 65 total host cellular proteins were identified as hantavirus interacting partners: 47 were associated with the PUUV NSs protein, while 21 were found to interact with the TULV NSs protein. The overlap between the two screens comprised three proteins: Acyl-coenzyme A-binding domain containing 3 protein (ACBD3), ARP5 actin-related protein 5 homolog (ACTR5), and keratin-14 (KRT14). ACBD3 was validated as an interacting partner of TULV NSs by fluorescence resonance energy transfer (FRET) and colocalization of the two proteins in the perinuclear area was observed by confocal microscopy. Bioinformatic analysis of the pooled interactome of 65 proteins revealed overlapping cellular pathways between the two hantavirus NSs proteins. This dataset provided insight into potential, previously-undescribed roles for NSs during infection, including regulation of apoptosis and interaction with proteins of the integrin complex. 

An extensive survey of viral-host protein-protein interactions by Pichlmair *et al.* in 2012 used as bait a panel of 70 viral open reading frames (ORFs) selected for their roles in defending against the host innate immune response [37]. The bunyavirus ORFs included in the panel were the NSs of RVFV, LACV, and Sandfly fever Sicilian virus (SFSV). Viral ORFs were expressed within a HEK293 cell line and then affinity purified followed by liquid chromatography tandem-mass spectrometry (LC-MS/MS). The authors identified 579 interacting host proteins, which displayed an overrepresentation of proteins known to be involved in innate immunity, and specifically they noted an enrichment within the interacting partners of the negative-sense single-strand RNA for host proteins that may promote processing of viral RNA transcripts or prevent detection and degradation of these transcripts. In 2014, a follow-up study was published by Kainulainen *et al.* examining the interaction between RVFV NSs and the host F-box protein FBXO3 [38]. FBXO3, which is a component of an E3 ubiquitin ligase, was shown to be recruited by NSs to effect the degradation of p62, a subunit of the general transcription factor II Human (TFIIH). Depletion of FBXO3 failed to fully rescue interferon induction in RVFV-infected cells, did not affect the ability of NSs to degrade the interferon-induced antiviral effector dsRNA-dependent protein kinase R (PKR), and did not significantly impact viral replication. The authors therefore concluded that this FBXO3-mediated degradation of p62 is partially, although not completely, responsible for the ability of NSs to suppress the host interferon response. These findings highlight the capacity of protein-protein interaction studies for uncovering host factors that might not have been detected by gene-disruption or gene-depletion screening strategies, which usually depend upon robust viral replication or host cell survival phenotypes.

## 6. The Next Generation of Biochemical Screening Techniques

To identify potential cellular interacting partners during bunyavirus infection, it is now possible to circumvent the requirement that proteins associate strongly with bait proteins during affinity purification. Martell *et al.* introduced in 2012 a new genetically-encoded reporter molecule that can be used for proximity labeling followed by MS to detect nearby proteins, as well as electron microscopy [39,40]. The authors engineered a monomeric variant of ascorbate peroxidase, which they have termed APEX, that is active in all cellular compartments (including the cytosol), a major advantage over the horseradish peroxidase (HRP) tag typically used. This APEX tag can oxidize biotin-phenol (in the presence of a hydrogen peroxide catalyst) into phenoxyl radicals, and these short-lived radical species react with electron-rich amino acids present in proteins that are fewer than 20 nm away. This results in the biotin-labeling of endogenous proteins adjacent to the APEX-tagged protein of interest, and these can be identified by streptavidin purification followed by digestion and MS analysis. An improved version of this peroxidase, termed APEX2, was recently obtained by yeast display evolution and exhibits increased activity, stability, and sensitivity [41].

Another proximity labeling approach developed in 2012 by Roux and colleagues is named proximity-dependent biotin identification (BioID) and it employs a promiscuous mutant of the *E. coli* biotin ligase BirA fused to a bait protein of interest [42]. As with the APEX labeling technique, neighboring proteins that have been biotinylated within the cell can be affinity purified and identified. BioID has been used to characterize the constituents and architecture of the nuclear pore complex and to identify the interactome of the Ewing sarcoma fusion oncoprotein EWS-Fli-1 [43,44]. This approach has also been used to study host-pathogen interactions during bacterial and viral infection. Mojica *et al.* fused the BirA to SINC, a type III secreted effector from *Chlamydia psittaci*, and showed that it targets the nuclear envelope of both infected and neighboring cells [45]. In 2015, Le Sage and colleagues used HIV-1 Gag protein fused to BirA to identify 47 associated proteins that were biotinylated by the fusion protein when it was transfected into Jurkat cells [46]. Two of the putative host factors identified, DDX17 and RPS6, were validated as interacting partners of Gag by co-immunoprecipitation experiments. A substantially smaller biotin ligase, BioID2, was recently described to have higher activity and to improve the function and localization of the resultant fusion protein [47]. These new proximity labeling technologies represent exciting additions to the bunyavirus screening toolbox. 

**Figure 1:**
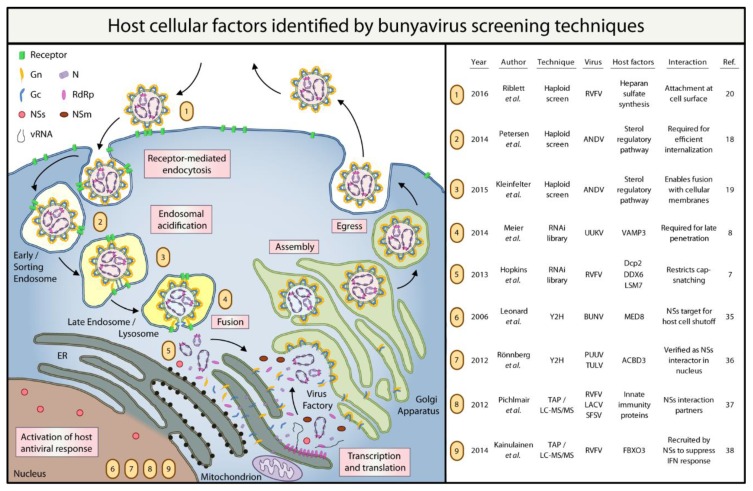
Summary of bunyavirus host factors identified by high-throughput screening techniques. RdRp: RNA-dependent RNA polymerase; NSs: non-structural protein S; NSm: non-structural protein M; vRNA: viral RNA; RVFV: Rift Valley fever virus; ANDV: Andes virus; UUKV: Uukuniemi virus; BUNV: Bunyamwera virus; PUUV: Puumala virus; TULV: Tula virus; LACV: La Crosse encephalitis virus; SFSV: Sandfly fever Sicilian virus; VAMP3: vesicle-associated membrane protein 3; Dcp2: mRNA-decapping enzyme 2; DDX6: DEAD (Asp-Glu-Ala-Asp) Box Helicase 6; LSM7: U6 snRNA-associated Sm-like protein LSm7; MED8: Mediator of RNA polymerase II transcription subunit 8; ACBD3: acyl-coenzyme A-binding domain containing 3 protein; FBXO3: F-Box Protein.

Please refer to Figure 1 for a summary of the host cellular factors that have been identified by the bunyavirus screens discussed in this review.

## 7. Diverse Screening Approaches are Highly Complementary

As RNAi screening became more popular, it also became evident that the technology suffered from issues of reproducibility and a high rate of false discovery. Results of the three genome-wide siRNA screens performed with HIV in 2008 [3,4,5], each of which had generated a list of approximately 300 genes supporting HIV infection in 293T or HeLa-derived cells, were subjected to in-depth meta-analysis by Bushman *et al.* in 2009, who reported that the percentage of overlap in gene hits between any two of the three screens was 6% at most [48]. Two genome-wide RNAi screens were performed in 2009 to uncover host factors required for hepatitis C virus (HCV) replication in human cells. Tai *et al.* [49], using an HCV subgenomic replicon, reported the identification of 96 genes that support HCV replication, and Li *et al.* [50], using infectious virus, then identified 262 genes impacting infection, only 15 of which overlapped with the previous screen’s findings. In the last five years, two genome-wide RNAi screens using Sindbis virus (SINV) have been performed, one in *Drosophila* cells [51,52], and one in human cells [53]. The screen in *Drosophila* cells identified 57 genes supporting and 37 genes that restricted SINV infection, while the screen in human cells identified 56 genes supporting and 62 genes restricting infection—but there was very little overlap between the genes identified (compare [53] Tables S2 and S3 to human homologues of [52] Table S1).

Much of the reason for this lack of overlap between seemingly similar RNAi screens has been ascribed to the off-target effects of siRNAs and differences between technical aspects of the screening conditions. In a recent analysis of three genome-wide RNAi screens (one with UUKV and two with bacterial pathogens), Franceschini *et al.* concluded that the phenotypic effects of siRNA oligos were in fact predominantly due to off-target microRNA activity conferred by the seed region sequence, rather than the intended siRNA activity [54]. They found significantly higher phenotypic correlations when siRNA oligos from different vendors were grouped by seed sequence (nucleotides 2–8) than when they were grouped by intended target (full-length complementarity of all 21 nucleotides). The authors confirmed these findings by designing custom oligos containing seed sequences predicted to impact infection that were flanked by arbitrary sequences outside of the seed region, and demonstrated that overexpression of known human microRNAs phenocopied the effect of siRNA oligos with corresponding seed sequences. These findings beg a reexamination of the raw data that have been generated by previous RNAi screens, as well as an attentive consideration of microRNA effects during analysis of any future screens. In addition to the off-target activities of the oligos themselves (which can cause both false-positive and -negative results), differing gene expression levels between cell types, variable efficiencies of transfection protocols, and discordance between knockdown timing and the half-life of the target protein can all contribute to a high false-negative rate. Recent improvements in both design and analysis of RNAi screens have sought to address these problems, such as the Minimum Information About an RNAi Experiment (MIARE) reporting guidelines (http://miare.sourceforge.net) that have been established, and the utilization of the multiple orthologous RNAi reagents coupled with RNAi gene enrichment ranking (MORR-RIGER) method, which helps to reduce false negatives and filter off-target effects [55]. For a detailed discussion about the factors impacting RNAi screen success, recent technical updates, and current design and analysis strategies, see reference [56] and the references therein.

Like RNAi, insertional mutagenesis screening is a forward genetic approach, allowing for discovery of novel host factors in the absence of a presumed or suspected mechanism of action. Although the technique is relatively new and comparatively few studies employing this approach to study virus-host interactions have been published, it is clear that haploid screening offers some important advantages over RNAi screening. A significant advantage of haploid screening is the fact that the insertional mutagenesis strategy employed to generate the haploid libraries usually results in complete disruption of the gene product, rather than the transient partial depletion that results from RNAi targeting. This in turn greatly increases the signal-to-noise ratio of the data that are obtained. Generation of many independent mutants within the library that each bear separate integrations into the same gene locus also allows for rigorous statistical analysis to identify genes whose absence was selected for within the surviving mutant pool. The fact that this selection occurs in a cell line of human origin is also attractive because it increases the likelihood of finding biologically meaningful factors that participate in the host-pathogen interaction during the course of human disease.

It may be premature to evaluate the reproducibility of haploid genetic screens as published applications of this screening technique have utilized a diverse array of viruses and toxins, including EBOV, LASV, RVFV, enterovirus D68, and adeno-associated virus serotype 2 [15,16,17,20,57,58]. In addition, diphtheria and anthrax toxins, *Clostridium perfringens* TpeL toxin, *Pseudomonas aeruginosa* exotoxin A, and *Staphylococcus aureus* α-toxin [13,59,60,61] have been investigated with this approach. To our knowledge, ANDV is the only selective agent to have been used in two completely independent haploid genetic screens performed by different labs. The degree of overlap between these two screens, however, was striking. In the 2014 study by Petersen *et al.* four genes encoding members of the sterol regulatory pathway (*SREBF2*, *S1P*, *S2P*, and *SCAP*) were enriched for disrupting integrations well above any other genes [18] and the 2015 screen performed by Kleinfelter *et al.* reported that these exact same four genes were also their top hits, and that three other genes involved in cholesterol biosynthesis (*LSS*, *SQLE*, and *ACAT2*) were the next most frequently disrupted [19]. This identification of multiple members of a biological pathway has been seen in many of the haploid screens mentioned in this review, and it not only demonstrates the high level of mutagenesis coverage in the libraries that have been generated thus far, but it also increases the confidence that screening hits are biologically relevant.

The haploid screening technique is not without drawbacks. Due to the nature of disrupting mutagenesis in a haploid genetic background, this screening strategy is unlikely to identify host factors that are required for cell viability. Additionally, most haploid screens have relied upon cell death as a phenotypic read-out, a decision that greatly increases the throughput of the screen but that may prevent the identification of a gene whose disruption produces an intermediate phenotype in which virus infection is delayed or partially suppressed. We find it interesting that in a number of the published screens a single biological pathway is clearly identified by virtue of multiple retroviral gene insertions to the near exclusion of other hits. In the two ANDV haploid screens [18,19], cells that survived the viral challenge almost invariably had one of several genes involved in cholesterol biosynthesis disrupted, and in the RVFV haploid cell screen we performed, genes contributing to glycosaminoglycan synthesis and Golgi complex function were mutated in the surviving pool almost to the exclusion of any other mutations. In contrast, RNAi screens often implicate several biological pathways as being important for viral replication, as did the RVFV RNAi screen published by Hopkins *et al.* [7]. Variables that could impact the results of haploid cell screens could include the multiplicity and timing of infection as well as the length of time cells are cultured after virus challenge. Finally, most haploid screens have utilized mutant libraries generated in the human haploid cells HAP1, a line derived from the KBM-7 chronic myeloid leukemia cell line, which restricts its use to viruses that are capable of infecting these cells. 

Interrogating host-pathogen protein-protein interactions through Y2H, AP/MS, or proximity labeling makes it possible to identify host factors based upon the *a priori* association of a viral and a cellular protein within the biological context of the host cellular environment. Many of the common phenotypic read-outs used during screening techniques, such as production of a reporter protein or host cell death, have the distinct disadvantage of restricting host factor discovery to those which impact a specific subset of stages during the viral replication cycle. High-throughput screens to identify cellular factors required for viral assembly and egress, for example, have proven difficult to design and, screens to identify host factors required for viral infections have largely focused on the rate-limiting stages of entry and replication. Another important advantage to protein-protein interaction screening is that it allows for the identification of host factors whose depletion or disruption may be cytotoxic, or even lethal. On the other hand, antibodies to affinity purify a viral protein are not always available, and the introduction of a tag or the precipitation conditions may perturb viral protein function or have other unforeseen consequences.

The use of multiple complementary screening techniques can serve to address and overcome the varying advantages and disadvantages presented by each of the techniques on their own. Performing multiple screens in parallel can help eliminate false-positive hits, even if the differences between the screens are relatively subtle technical changes such as use of different viral strains, cell types, or siRNA libraries. With each new published screen, the pool of datasets available to draw from also increases, which will allow for valuable comparisons of one’s screening results with the reported hits from other related screens. 

## 8. Future Perspectives: Expanding Cellular Targets and Bunyavirus Technical Resources

In addition to the screening techniques focused on genes and proteins, there has been renewed interest in developing high-throughput approaches to identify metabolites and lipids that are involved in viral infection. Analysis with LC-MS can be used to quantify changes in the metabolomic profile of infected cells relative to uninfected cells, providing insight into viral alteration of host metabolism as well as yielding potential therapeutic targets. This approach was used to quantify the levels of known metabolites at different time points during infection with human cytomegalovirus (HCMV), herpes simplex virus type-1 (HSV-1), and IAV, demonstrating each virus’s ability to differentially remodel the host’s metabolism during infection [62,63,64]. In the case of HCMV and IAV, pharmacological inhibition of fatty acid biosynthesis was shown to effectively restrict viral replication, demonstrating the power of such screens to inform the development (or repurposing) of therapeutics. In 2013, Morita *et al.* tested a library of bioactive lipids for an effect on IAV replication, and observed potent inhibition with the lipid mediator protectin D1 (PD1) [65]. Treatment with PD1 was able to protect against influenza in a mouse model, even if it was not supplied until severe disease had developed. Another important aspect of virus-host dynamics that could be examined for bunyaviruses is that of interactions between RNA and proteins during infection. Yeast three-hybrid (Y3H) screening provides a powerful tool for identifying proteins that bind to a specific RNA sequence. This technique, first described by SenGupta *et al.* [66], detects RNA-protein interactions by utilizing two-hybrid proteins whose proximity activates a reporter gene when both proteins bind to a hybrid RNA molecule. Y3H screening was used to identify human ribosomal proteins that bind to the 3’ untranslated region of HCV using a human cDNA library as prey and the viral RNA sequence as bait [67]. Covalent UV crosslinking during infection could also be used to capture and characterize the RNA-protein interactome in a manner similar to the technique described by Castello *et al.* in 2012 [68].

These and other recent advances in screening technology have the potential to significantly inform bunyavirus research, particularly in light of the continually expanding options available for generating bunyavirus reporter systems to enable high-throughput or automated screening. Among orthobunyaviruses, a replication-competent recombinant BUNV has been generated bearing a fluorescent or V5 tag on either Gc glycoprotein or L proteins, respectively [69,70]. In 2013, reverse genetics was described for Schmallenberg virus (SBV) and in 2015 a BHK cell line was developed that constitutively expressed the SBV N protein and a minigenome system was described for Oropouche virus (OROV) [71,72,73]. Efficient reverse genetics has also now been established for Akabane virus (AKAV), further expanding the options for bunyavirus screening approaches [74]. For the phlebovirus RVFV, there exists both a reverse genetics toolset as well as a BHK replicon cell line expressing the S and L segments of the genome [75,76]. We and others have also utilized pseudovirion systems, described in [77] and [78], in order to screen for host factors required during entry of bunyaviruses. These pseudotyped virions can be used at the biosafety level (BSL)-2 and allow for the convenient use of either cell death or a genetically-encoded reporter (e.g., luciferase or a fluorescent protein) to facilitate high-throughput, cell-based screening approaches. The future of bunyavirus screening techniques is bright, and the marriage of improved screening techniques with the increasing availability of virological tools promises to push forward our understanding of how these viruses interact with their host cells, and will help us develop targeted antiviral therapeutics.
